# Anti-inflammatory effects of novel curcumin analogs in experimental acute lung injury

**DOI:** 10.1186/s12931-015-0199-1

**Published:** 2015-03-24

**Authors:** Yali Zhang, Dandan Liang, Lili Dong, Xiangting Ge, Fengli Xu, Wenbo Chen, Yuanrong Dai, Huameng Li, Peng Zou, Shulin Yang, Guang Liang

**Affiliations:** Chemical Biology Research Center at School of Pharmaceutical Sciences, Wenzhou Medical University, Wenzhou, Zhejiang 325035 China; School of Environmental and Biological Engineering, Nanjing University of Science and Technology, Nanjing, Jiangsu 210094 China; The 2nd Affiliated Hospital, Wenzhou Medical University, Wenzhou, Zhejiang 325035 China

**Keywords:** Curcumin, LPS, Acute lung injury, ERK

## Abstract

**Background:**

Acute lung injury (ALI) and its most severe form acute respiratory distress syndrome (ARDS) have been the leading cause of morbidity and mortality in intensive care units (ICU). Currently, there is no effective pharmacological treatment for acute lung injury. Curcumin, extracted from turmeric, exhibits broad anti-inflammatory properties through down-regulating inflammatory cytokines. However, the instability of curcumin limits its clinical application.

**Methods:**

A series of new curcumin analogs were synthesized and screened for their inhibitory effects on the production of TNF-α and IL-6 in mouse peritoneal macrophages by ELISA. The evaluation of stability and mechanism of active compounds was determined using UV-assay and Western Blot, respectively. *In vivo*, SD rats were pretreatment with **c26** for seven days and then intratracheally injected with LPS to induce ALI. Pulmonary edema, protein concentration in BALF, injury of lung tissue, inflammatory cytokines in serum and BALF, inflammatory cell infiltration, inflammatory cytokines mRNA expression, and MAPKs phosphorylation were analyzed. We also measured the inflammatory gene expression in human pulmonary epithelial cells.

**Results:**

In the study, we synthesized 30 curcumin analogs. The bioscreeening assay showed that most compounds inhibited LPS-induced production of TNF-α and IL-6. The active compounds, **a17**, **a18**, **c9** and **c26**, exhibited their anti-inflammatory activity in a dose-dependent manner and exhibited greater stability than curcumin *in vitro*. Furthermore, the active compound **c26** dose-dependently inhibited ERK phosphorylation. *In vivo*, LPS significantly increased protein concentration and number of inflammatory cells in BALF, pulmonary edema, pathological changes of lung tissue, inflammatory cytokines in serum and BALF, macrophage infiltration, inflammatory gene expression, and MAPKs phosphorylation . However, pretreatment with **c26** attenuated the LPS induced increase through ERK pathway *in vivo.* Meanwhile, compound **c26** reduced the LPS-induced inflammatory gene expression in human pulmonary epithelial cells.

**Conclusions:**

These results suggest that the novel curcumin analog **c26** has remarkable protective effects on LPS-induced ALI in rat. These effects may be related to its ability to suppress production of inflammatory cytokines through ERK pathway. Compound **c26**, with improved chemical stability and bioactivity, may have the potential to be further developed into an anti-inflammatory candidate for the prevention and treatment of ALI.

**Electronic supplementary material:**

The online version of this article (doi:10.1186/s12931-015-0199-1) contains supplementary material, which is available to authorized users.

## Introduction

Acute lung injury (ALI) is defined as acute inflammatory lung injuries associated with histopathological changes including neutrophilic alveolar infiltrates, impaired alveolar fluid clearance, fibrin deposition and lung edema. Despite advances in therapies, the outcomes of ALI in critically ill patients remain dismal with a morbidity and mortality rate around 40% [1-3]. With improved understanding of the pathogenesis of ALI, accumulating evidence shows that the release of pro-inflammatory cytokines play a critical role in inflammation-induced lung injury. Previous reports indicated that tumor necrosis factor (TNF)­α, interleukin (IL)­6, IL-1β, and IL­8 are the key inflammatory mediators involved in the progression of ALI [4-6].

Numerous pharmacological agents were investigated in an effort to attenuate the release of these pro-inflammatory cytokines involved in ALI. In pre-clinical experiments, these anti-inflammatory agents have demonstrated potent inhibitory effects on the release of inflammatory mediators and protective effects on ALI [7-10]. However, several pharmacological therapeutic trials failed to demonstrate any benefit in patients with ALI [11,12]. The failure of prior clinical trials of several pharmacological agents may be partly due to the delay of therapy which occurred several days after the onset of ALI. Therefore, pharmacological therapies for prevention or early intervention of ALI have emerged as a new paradigm [3]. Some of the most promising therapeutic agents for early treatment of ALI include aspirin, statins, beta-2 adrenergic agonists, corticosteroids, vitamin D, and butyrate [3,13-15].

Curcumin, a natural product isolated from turmeric, has been found to have broad anti-inflammatory activities both *in vitro* and *in vivo.* However, the poor solubility and chemical instability of curcumin, under physiological conditions, limit its bioavailability and clinical efficacy [16-18]. Curcumin analogs have been designed to improve bioavailability and bioactivity. Among them, mono-carbonyl analogs of curcumin (MACs) demonstrate excellent chemical stability and pharmacokinetic profiles [19-21]. We previously synthesized and identified a mono-carbonyl analog of cucurmin (C66), which demonstrated excellent chemical stability and potent anti-inflammatory effects both *in vivo* and *in vitro* [22,23]. Recent studies indicate that curcumin has potential protective effects for ALI [24-26]. However, there is no report on the effects of curcumin analogs on lipopolysaccharide (LPS)-induced ALI. We considered that the investigation of the effects of novel curcumin analogs with improved chemical stability may discover novel anti-inflammatory candidate agents for the prevention or treatment of ALI.

## Materials and methods

### Animals and reagents

Male ICR mice (6 wk, 18-20 g) and Sprague–Dawley (SD) rats (6 wk, 180-200 g,) were obtained from Shanghai SLAC Laboratory Animal Center, CAS (SLACCAS). Mice were housed under specific pathogen-free conditions with a 12-hour/12-hour light–dark cycle and maintained on a normal diet at Wenzhou Medical University Animal Center. All mice and *in vivo* experiments were performed in accordance with procedures approved by Wenzhou Medical University Animal Policy and Welfare Committee (Approval Documents: 2013/APWC/0361).

LPS was purchased from Sigma (*St. Louis, MO*). Enzyme-linked immunosorbent assay (ELISA) kits of TNF-α and IL-6 were obtained from eBioscience, Inc. (San Diego, CA, USA). Extracellular signal-regulated kinase (ERK), p-ERK and CD68 antibodies were purchased from Santa Cruz Biotechnology, Inc., (Santa Cruz, CA, USA). Antibodies of p-P38, P38, p-Jun N-terminal kinase (JNK), and JNK were obtained from Cell Signaling Technology, Inc., (Danvers, MA, USA).

### Harvest and culture of mouse primary peritoneal macrophages (MPMs)

ICR mice were stimulated by intraperitoneal injection of 6% thioglycolate broth (0.3 g beef extract, 1 g tryptone, 0.5 g NaCl and 6 g starch dissolved in 100 mL H_2_O, 1.5 mL/mouse) for 3 days before sacrificed for MPMs harvest. MPMs were then centrifuged and suspended in RPMI-1640 medium (Gibco/BRL life Technologies, Eggenstein, Germany) supplemented with 10% FBS and 1% penicillin/streptomycin. The cells were incubated overnight at 37°C in a 5% CO_2_-humidified air.

### Detection of TNF-α and IL-6 expression by ELISA

The MPMs harvested were pre-treated with curcumin (10 μM), compounds (10 μM), or DMSO (control) for 30 minutes, which was followed by the treatment of 0.5 μg/mL LPS. After treatment, the cells were incubated for 24 hours. The media were collected to measure the amount of TNF-α and IL-6 through the use of ELISA kit (eBioScience, San Diego, CA) according to manufacturer’s protocol. The total protein concentrations in viable cell pellets were measured. The amounts of TNF-α and IL-6 were normalized to the total proteins in cells.

### Chemical stability analysis of curcumin analogs by UV absorbance spectroscopy

Curcumin or the active compounds were dissolved in DMSO (1 mM) and diluted with PBS (pH 7.4) to 20 μM. The absorbance spectra were taken from 250 to 600 nm at 25°C on SpectraMax® M5 (Molecular Devices LLC, Sunnyvale, CA, USA). Absorbance spectral readings were recorded for over a time span of 25 minutes at 5 minute intervals.

### Western blot analysis

The macrophages harvested from mice were pretreated with the active compounds or DMSO (control) for 30 minutes and then incubated with 0.5 μg/mL LPS for 20 minutes. After treatment, protein samples were collected, separated by SDS-PAGE, and transferred to PVDF membranes. Membranes were incubated with a blocking solution of 5% non-fat milk for 1.5 h at room temperature. Proteins on membranes were then separately probed with the primary antibodies overnight. Protein samples were further incubated with horseradish peroxidase-conjugated (HRP) secondary antibodies for 1 h, and visualized using enhanced chemiluminescence reagents (Bio-Rad Laboratories).

### Animal models of ALI

SD rats were randomly placed into three groups (n = 6, each group). The rats were anesthetized and the tracheas were surgically exposed. Group 1 (CON group) received an intratracheal injection of saline. Group 2 (LPS group) received a drop-wise intratracheal injection of LPS (5 mg/kg, 50 μL), Group 3 (**CUR** + LPS group) and Group 4 (**c26** + LPS group) received an intra-gastric administration of compound **c26** (20 mg/kg/day) daily for seven days prior to the administration of LPS (50 μL, 5 mg/kg). Six hours after LPS administration, all animals were anesthetized by chloral hydrate and sacrificed. Broncho-alveolar lavage fluids (BALF) were collected for determination of total protein concentration and inflammatory cell infiltration. The lobes of the right lung were harvested for the study of curcumin and **c26** on LPS induced lung injury analysis.

### BALF

Thoracotomy and ligation of the left lung were performed. The left lung was infused three times with 1 mL phosphate buffered saline (PBS) in order to obtain BALF as previously described [27]. The collected BALF was centrifuged for 10 minutes at 1,000 rpm. Cell-free supernatant was used for measurement of target protein cytokines. The cell pellets obtained from BALF were washed and re-suspended in 50 μL PBS for cell counting with a Hemocytometer.

### Lung wet/dry weight ratio

The right upper lobe of lung was excised. After removal of the excessive water on the tissue surface, the wet weight was recorded. The sample was then dried at 60°C for 48 h until no weight change to record the dry weight. The wet weight/dry weight ratio (W/D) was calculated and used as an index of lung edema.

### Pulmonary histopathology and immunohistochemistry analysis

The right lower lobe of lung was excised and fixed with 4% formalin. The lung tissues were embedded with paraffin, sliced to 5 μm sections, and stained with hematoxylin and eosin (HE). Rat lung histopathology images were acquired using a microscope (Nikon Model Eclipse 80i, Nikon, Tokyo, Japan). The immunohistochemistry analysis was performed following the anti-CD68 antibody staining protocol.

### Histological analysis

The lung injury was evaluated by Lung injury scoresas described previously [28]. In brief, no injury = score of 0; injury in 25% of the field = score of 1; injury in 50% of the field = score of 2; injury in 75% of the field = score of 3; and injury through out the field = score of 4. Ten random microscopic fields from each slide were analyzed. The average score of the 10 slides was used to assess the severity of lung injury.

### Real-time PCR analysis for mRNA expressions of TNF-α, IL-6, IL-1β and COX-2

Total RNA was isolated from Beas-2B cells or lung tissue of rats with ALI using Trizol, then reversely transcribed to cDNA using M-MLV according to guidelines of the manufacturer. Gene specific primers used for TNF-α, IL-6, IL-1β, COX-2 and β-actin are listed in Additional file 1: Table S1.

### Statistical analysis

Data collected from experiments were analyzed using Graphpad prism 5.0 software. Values were expressed as mean ± SEM. One way ANOVA test was employed to analyze the differences between sets of data. A p-value of < 0.05 was considered as statistically significant and denoted as*. *In vitro* experiments were performed with n ≥ 3 independent repeats. *In vivo* experiments were performed with n ≥ 5 rats in each group.

## Results

### Chemistry

**Scheme 1:**
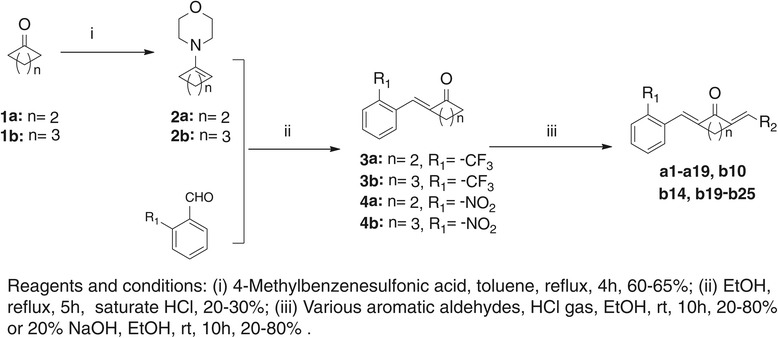
**The synthetic pathway of curcumin analogs.**

**Scheme 2:**
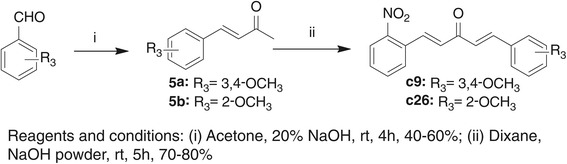
**The synthetic pathway of curcumin analogs c9 and c26.**

**Figure 1:**
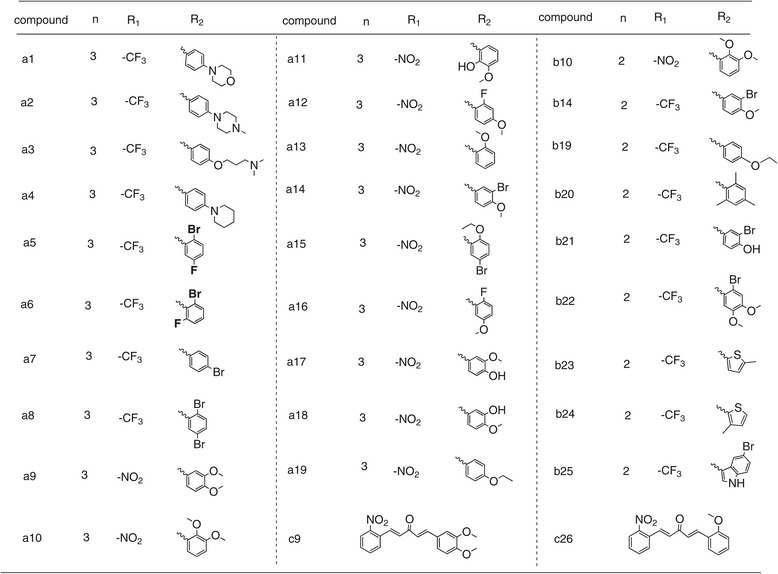
**The structures of synthesized curcumin analogs.**

In the present study, curcumin analogs were obtained from the Claisen–Schmidt condensation of substituted 3-phenyl-ketone and various aromatic aldehydes. The synthetic roots were listed in Scheme 1 and Scheme 2 and the chemical structures of these analogs were shown in Figure 1. Reaction progress was monitored by thin layer chromatography (TLC) and the structures of compounds were confirmed by ESI-MS and ^1^H-NMR. The detailed synthetic routes, synthetic yields, melting points, ^1^H-NMR, and ESI-MS analysis of novel and unpublished compounds are being described in Additional file 2.

### Screening of curcumin analogs for inhibition of the expression of cytokines TNF-α and IL-6

**Figure 2:**
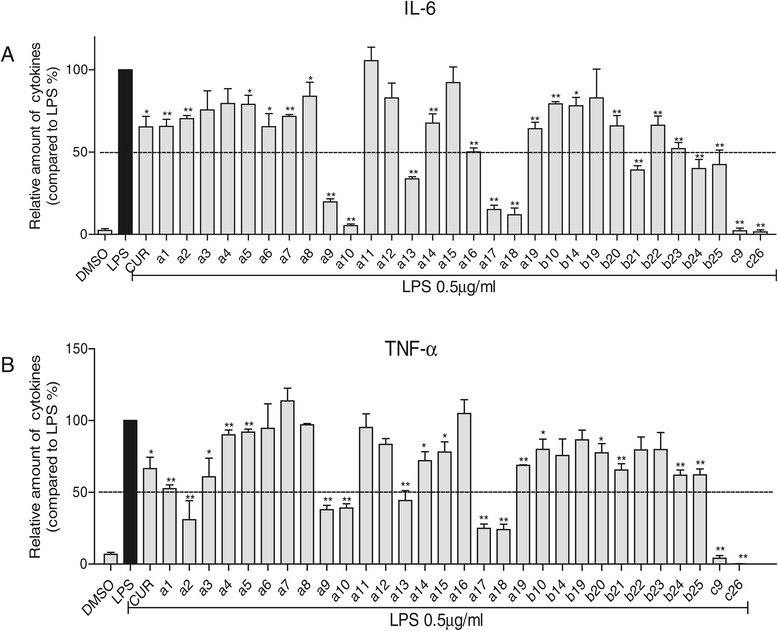
**Production of LPS-induced inflammatory cytokines in mouse peritoneal macrophages after treatment with curcumin derivatives.** Macrophages were treated with vehicle or 10 μM curcumin derivatives or 10 μM curcumin for 30 minutes, and the LPS (0.5 μg/mL) was added to incubate for further 24 hours. The culture media was collected and the inflammatory cytokines, IL-6 **(A)** and TNF-α **(B)**, in media were detected by ELISA and normalized by total protein concentration. The results were presented as the percent of LPS control. Each bar represents mean ± SEM of three independent experiments. Statistical significance relative to LPS group was indicated, *p < 0.05, **p < 0.01.

As shown in Figure 2A and B, the expressions of TNF-α and IL-6 were significantly increased in mice macrophages with the treatment of LPS. Some of the analogs showed stronger activity than the leading compound, curcumin, for inhibiting the production of TNF-α and IL-6. Pretreatment with compound **a9, a10, a17, a18, c9** or **c26** (10 μM) for 30 minutes significantly suppressed the release of IL-6 with an inhibition rate of above 80% (Figure 2A). In addition, compounds **a2, a9, a10, a13, a17, a18, c9** or **c26** (10 μM) markedly attenuated the expression of TNF-α (Figure 2B). Compounds **a17, a18, c9** and **c26** efficiently inhibited the expression of both TNF-α and IL-6. The most potent compounds, **c9** and **c26,** inhibited virtually all releases of TNF-α and IL-6 in MPMs stimulated by LPS. The results from Figure 2 show that compounds, substituted by –NO_2_ on R_1_, exhibited stronger inhibitory activity than –CF_3_ on R_1_. Compared with cyclopentanone as the connecting link, cyclohexanone contributes to potent anti-inflammatory activity of these analogs. Moreover, the compound with R_2_ containing methoxyl and/or hydroxyl group on the phenyl ring showed better anti-inflammatory activities than their correspondence with the electron-withdrawing group.

### The effects of a17, a18, c9 and c26 on the expression of IL-6 and TNF-α

**Figure 3:**
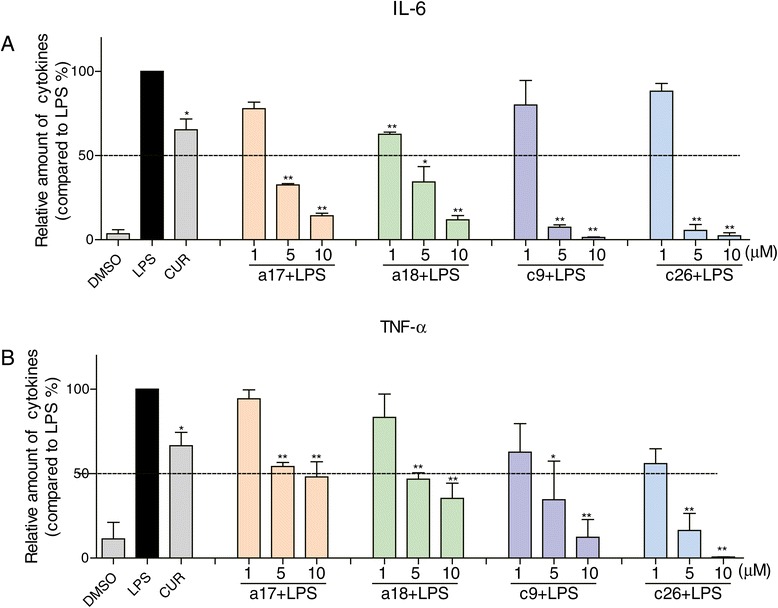
**Active compounds inhibited LPS-induced inflammatory cytokines in a dose-dependent manner.** Macrophages were treated with vehicle, 10 μM curcumin or (1, 5, 10 μM) active compounds for 30 minutes, and the LPS (0.5 μg/mL) was added to incubate for further 24 hours. The culture media was collected and the inflammatory cytokines IL-6 **(A)** and TNF-α **(B)** in media were detected by ELISA and normalized by total protein concentration. The results were presented as the percent of LPS control. Each bar represents mean ± SEM of three independent experiments. Statistical significance relative to LPS group was indicated, *p < 0.05, **p < 0.01.

The most active compounds, **a17**, **a18**, **c9** and **c26,** were further evaluated for their effects on the production of IL-6 and TNF-α in a dose-dependent manner. First, we evaluated the cytotoxicity of these active compounds using the MTT assay. The results shown in Additional file 3: Figure S1 and show no cytotoxicity. Then MPMs were pretreated with **a17**, **a18**, **c9** or **c26** in an escalating doses (1, 5, 10 μM), or DMSO (control) for 30 minutes, followed by the treatment of 0.5 μg/mL LPS. As shown in Figure 3, compounds **a17, a18, c9,** and **c26** significantly inhibited LPS-induced TNF-α and IL-6 release in a dose dependent manner. All four active compounds showed better inhibitory activity than curcumin even at 5 μM. The most potent compound, **c26** (5 μM), demonstrated potent inhibition (>90% inhibition rate) of IL-6 and TNF-α expressions induced by LPS. The results further validate that these compounds possess significant anti-inflammatory activity.

### Chemical stability analysis of bioactive compounds a17, a18, c9 and c26

**Figure 4:**
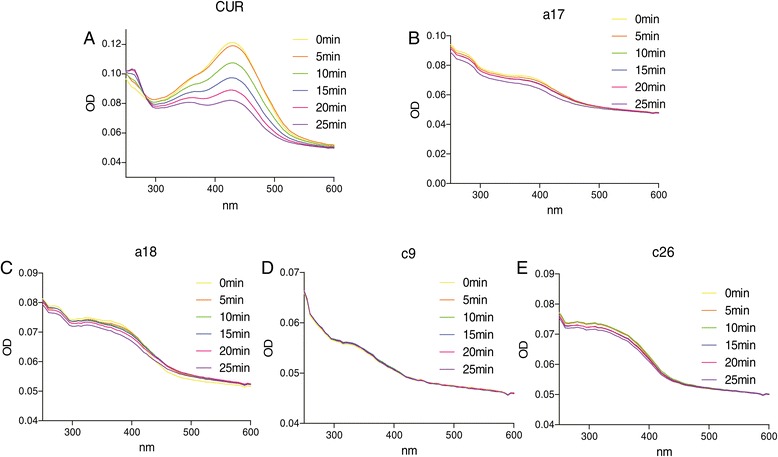
**UV-visible absorption spectra of curcumin, a17, a18, c9, and c26 in phosphate buffer (pH 7.4), containing 5% DMSO.** The compounds stability was described by the curve which is consisted of absorbance at various optical density (250–600 nm) and intervals (0, 5, 10, 15, 20, 25 min). **(A)** curcumin; **(B)** a17; **(C)** a18; **(D)** c9; **(E)** c26.

Previous report showed that 90% of curcumin rapidly decomposes in the physiological buffer solution (pH 7.4) which limits its bioavailability in clinical application [29]. To examine the chemical stability of these bioactive compounds, UV absorption spectra of bioactive compounds **a17**, **a18**, **c9,** and **c26** in PBS (5% DMSO and pH 7.4) were measured at different time points (0–25 min). Figure 4 shows that no significant changes in absorbance were observed for these bioactive compounds over the time. As a comparison, the UV peak absorbance of curcumin decreased significantly within 25 minutes as a result of chemical degradation. Meanwhile,we used the UV assay to detect the stability of these compounds after a longer period of incubation in PBS at 37°C (Data is shown in Additional file 3: Figure S2). The UV peak absorbance was disappeared after an incubation of 30 minutes, while the active compounds showed no obvious changes within 1 hour incubation. The results indicate that these novel curcumin analogs are chemically stable in the simulated physiological buffer solution (PBS, pH 7.4) as compared with curcumin.

### Effect of c26 on the phosphorylation of ERK, JNK, and p38 in MAPK pathways

**Figure 5:**
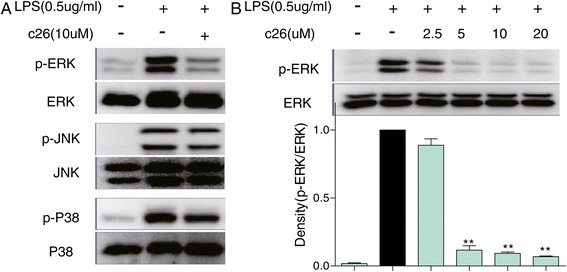
**Compound c26 inhibited ERK phosphorylation. (A)** Macrophages were cultured with or without **c26** for 30 min and stimulated with LPS (0.5 μg/mL) for an additional 20 minutes. Cells were harvested and the total protein was extracted. The protein level of p-ERK, ERK, p-JNK, JNK, p-P38, and P38 was determined by western blot, respectively. **(B)** Compound **c26** dose-dependently decreased ERK phosphorylation. Representative blots of three independent experiments in each study are shown. Statistical significance with regard to LPS was demonstrated, **P <0.01.

Previous studies have shown that multiple signaling pathways are involved in stimulating the releases of inflammatory cytokines. One of the critical signaling pathways is the Mitogen-activated protein kinase (MAPKs) pathway, which is comprised by ERK, c-Jun amino-terminal kinases (JNK), and p38. To investigate the possible mechanism by which the representative compound **c26** exerts its anti-inflammatory activity *in vitro*, we measured the effects of **c26** on phosphorylation of ERK, JNK, and p38. Figure 5A shows that the phosphorylation level of ERK (p-ERK), JNK (p-JNK), and p38 (p-p38) markedly increased after LPS treatment. Pre-treatment of **c26** (10 μM) for 30 minutes significantly inhibited the phosphorylation of ERK, however, it had little effect on the phosphorylation of JNK, and p38 (Figure 5A). Figure 5B also demonstrates that the active compound **c26** (2.5 ­ 20 μM) down-regulated the phosphorylation of ERK in a dose dependent manner.

### The effects of c26 on pulmonary histopathological changes of lungs in rats with ALI

**Figure 6:**
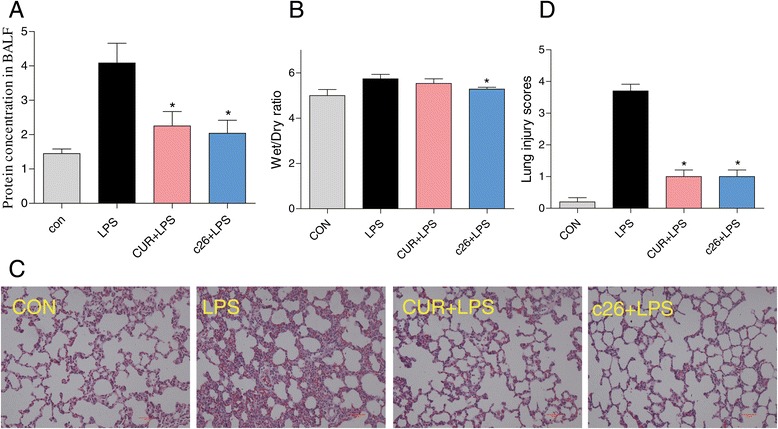
**Compound c26 attenuated lung pathophysiologic changes in LPS-challenged rats.** Effects of **c26** on the total protein concentration in **(A)** BALF and **(B)** the lung W/D ratio of LPS-induced ALI in rats. Statistical significance relative to LPS group was indicated, *p < 0.05. **(C)** Compound **c26** attenuated LPS-induced histopathological change in lung tissue (H&E staining). **(D)** The histogram of lung injury scores.

Gram-negative bacterial pneumonia and sepsis are major causes of clinical ALI. LPS, a component of the cell walls of gram-negative bacteria, was used to stimulate ALI in rat models *in vivo*. Figure 6A shows significant elevations of protein concentrations in BALF after LPS administration. The increased levels of protein concentrations in BALF were significantly reduced by administration of curcumin (CUR + LPS) or **c26** (**c26** + LPS). Lung wet/dry weight ratio is an index of lung edema. Figure 6B illustrates that LPS administration stimulated the increase of lung wet/dry weight ratio as compared with the control group. Pretreatment of **c26** (**c26** + LPS) reduced lung wet/dry weight ratio, while pretreatment with curcumin (CUR + LPS) showed no obvious effect. Figure 6C shows the effects of compound **c26** on the histopathological features of rat lungs with ALI. The control group shows normal structure of rat lung tissues. In the LPS group, remarkable alveolar wall thickness, hemorrhage, alveolar collapse, and inflammatory infiltration were observed in the lungs six hours after LPS administration (Figure 6C, middle panel). As a comparison, the CUR + LPS group and **c26** + LPS group show normal lung structure and very little histopathological changes. Figure 6D is the lung tissue injury scores correlated to Figure 6C. The results indicate that **c26** administration efficiently attenuated hemorrhage, alveolar collapse, and lung edema in rats with ALI.

### Effect of c26 on inflammatory cell infiltrations in rat lungs with ALI

**Figure 7:**
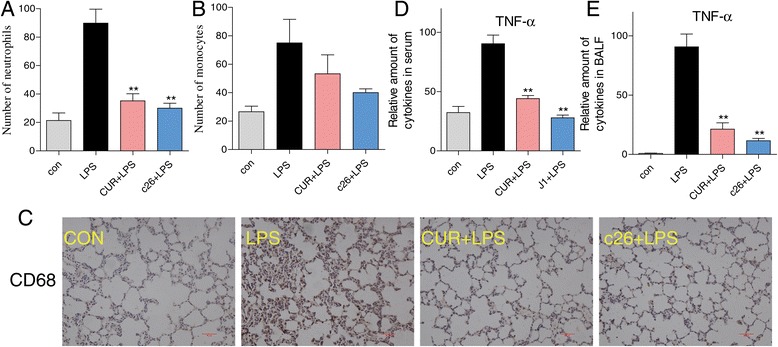
**Compound c26 attenuated lung inflammation in LPS-treated rats.** Compound **c26** reduced LPS-induced number of **(A)** neutrophils and **(B)** monocytes in BALF, **(C)** Compound **c26** reduced LPS-induced macrophages infiltration in lung tissue (CD68 immunostaining). Effect of c26 inhibited the inflammatory cytokine TNF-α expression in **(D)** serum and **(E)** BALF. Statistical significance relative to LPS group was indicated, *p < 0.05, **p < 0.01.

After onset of ALI, inflammatory cell infiltration in lung tissues and alveoli could further facilitate the inflammatory process associated with ALI. We examined the cell number in BALF, as shown in Figures 7A and 6B the neutrophil and monocyte numbers were markedly increased after LPS instillation. The **CUR** and **c26** reduced the LPS-induced inflammatory cells increase. To examine the effect of **c26** on macrophage infiltration in harvested rat lung tissue, we performed an immunohistochemical analysis with CD68 antibody. Rat lung tissue from the control group showed no macrophage infiltration (Figure 7C). In the LPS group, significant macrophage infiltration in rat lung tissue was observed. In the CUR + LPS group and the **c26** + LPS group, very minor macrophage filtration in lung was detected when compared to those in the LPS group (Figure 7C). Pro-inflammatory cytokines, including TNF-α, and IL-6, are major mediators involved in recruitment of neutrophils into the lungs in LPS-induced ALI. After LPS treatment, the concentration of TNF-α was increased both in serum and BALF. The response was significantly reduced by pre-treatment with curcumin or **c26**. Taken together, both curcumin and **c26** show obvious anti-inflammatory effects *in vivo.*

### The potential anti-inflammatory mechanism of c26 *in vivo*

**Figure 8:**
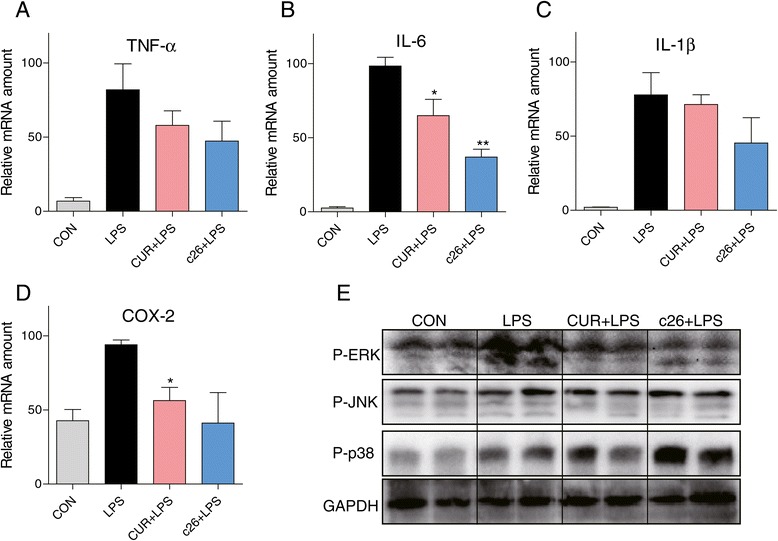
**Effects of c26 inhibited the LPS-induced inflammatory gene expression and inflammatory pathway in lung tissue. (A)** TNF-α, **(B)** IL-6, **(C)** IL-1β, and **(D)** COX-2 mRNA expression. **(E)** The phosphorylation of MAPKs. Statistical significance relative to LPS group was indicated, *p < 0.05, **p < 0.01.

Next, we measured whether pretreatment with the administration of **c26** could reduce the elevated mRNA expression levels of inflammatory cytokines in rat lung tissue. Significant increases in mRNA levels were observed for inflammatory mediators TNF-α (Figure 8A), IL-6 (Figure 8B), IL-1β (Figure 8C), and COX-2 (Figure 8D) after LPS administration. In contrast, administration of curcumin or **c26** markedly reduced mRNA expressions of those inflammatory mediators. Compound c26 performs its anti-inflammatory activity through the ERK pathway *in vitro. In vivo*, we detected the phosphorylation of MAPKs in lung tissue with ALI. Data from Figure 8E shows that curcumin and **c26** reduced the LPS instillation induced phosphorylation of ERK and had no effects on the phosphorylation of JNK and p38. These results were coincided with the data *in vitro*. These results indicate administration of **c26***in vivo* efficiently attenuated pulmonary inflammation in LPS-induced ALI by inhibiting the phosphorylation of ERK.

### Inhibition of IL-6 and IL-1β mRNA expression in human pulmonary epithelial cells

**Figure 9:**
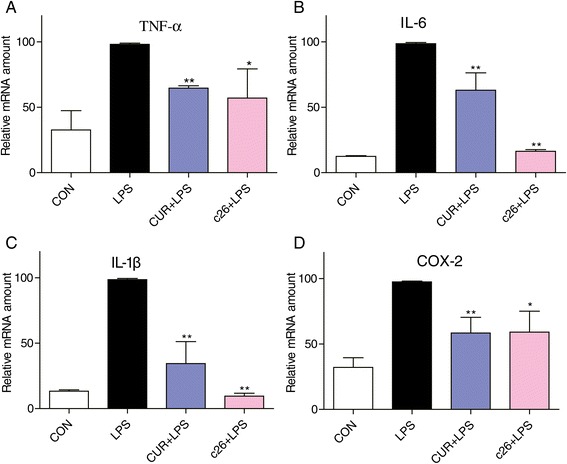
**Compound c26 reduced the LPS-induced inflammatory genes expression in human lung epithelial cells. (A)** TNF-α, **(B)** IL-6, **(C)** IL-1β, **(D)** COX-2. The results were presented as the percent of LPS control. Each bar represents mean ± SEM of three independent experiments. Statistical significance relative to LPS group was indicated, *p < 0.05, *p < 0.01.

We further examined the inhibitory effects of **c26** on LPS-stimulated mRNA expressions of TNF-α, IL-6, IL-1β and COX-2 in human pulmonary epithelial Beas-2B cells. Figure 9 shows that mRNA levels of pro-inflammatory cytokines, IL­6 and IL­1β, significantly increased after LPS stimulation for 12 h in Beas-2B cells. In contrast, pre-treatment with **c26** significantly suppressed the elevated mRNA expressions of IL­6 and IL­1β. In addition, **c26** attenuated the mRNA expressions of TNF-α and COX-2. within comparison to **c26**, curcumin showed a weaker effect on the inflammatory genes expression. The results observed in pulmonary epithelial cells *in vitro* are consistent with that of in rats with ALI *in vivo*. Collectively, these findings provide further evidence that lead compound **c26** could have a therapeutic effect on ALI by down-regulating the expression of the pro-inflammatory cytokine genes.

## Discussion

Acute lung injury (ALI) plays a pivotal role in the death of patients in intensive care unit. There is considerable experimental and clinical evidence indicates that inflammatory cytokines play a major role in the pathogenesis of LPS-induced lung injury [4,30]. A variety of new medications have appeared for the treatment of acute lung injury and new research on traditional therapies has been performed [31]. However, numerous pharmacological therapies for established ALI, including corticosteroids and steroids, have failed to show any therapeutic benefit in clinical trials [31,32].

TNF-α and IL-6 are important pro-inflammatory cytokines that can stimulate production of a host of other cytokines [33]. They have been measured to be elevated in ARDS BALF, however, measured values do not predict clinical outcome [34]. IL-6 gene-deficient mice are insensitive to pneumococcal pneumonia following intranasal inoculation of *Streptococcus pneumonia* [35]. Previous reports indicated that natural products have the ability to attenuate LPS-induced acute lung injury via down-regulation of TNF-α and IL-6 production [36-38]. In our study, the majority of the synthesized compounds had the ability to reduce LPS-induced TNF-α and IL-6 production (Figure 2). Furthermore, the active compounds **a17**, **a18**, **c9** and **c26** showed a dose-dependent inhibitory activity (Figure 3). These results demonstrated that the synthesized compounds have the potential to treat ALI by inhibiting inflammatory cytokines production.

After LPS incubation, TLR4 signaling was initiated, thus leading to the activation of NF-kB and MAPK [39]. NF-κB is a nuclear transcription factors that controls transcription of DNA [40]. Many researchers have reported that curcumin and curcumin analogs have the ability to inhibit the LPS-induced activation of NF-κB [21,41]. Unfortunately, the compounds in our research exhibited no effect on the activation of NF-κB (Data not shown). MAPK are a family of protein serine/threonine kinases that contain three subunits which include ERK, JNK, and P38 MAPK [42]. Increased activity of MAPK and their involvement in the regulation of the synthesis of inflammatory mediators at the level of transcription and translation, make them potential targets for anti-inflammatory therapeutics [43,44]. Zhan et al. reported that penehyclidine hydrochloride ameliorated acute lung injury through the inhibition of ERK1/2 and p38 MAPK activation in septic mice [45]. In this study, we found that LPS could activate all of three pathways of MAPKs (Figure 3A). Interestingly, compound **c26** exhibited no inhibitory ability on the phosphorylation of JNK and p38, however, it dose-dependently inhibited ERK phosphorylation induced by LPS (Figure 5). These results suggest that the inhibition of the production of TNF-α and IL-6 by **c26** may be mediated by the down-regulation of the ERK signaling. The underlying mechanism of the anti-inflammatory action of **c26** needs to be further investigated.

It has been reported that sepsis, especially gram negative bacteria infection, is the main cause of ALI/ARDS [46,47]. LPS, a major component of gram negative bacteria cell walls, was usually used to induce ALI in animals [48,49]. In our *in vivo* study, the model of acute lung injury was induced by intratracheal instillation LPS. LPS significantly increased protein concentration and the number of inflammatory cells in BALF, pulmonary edema, pulmonary histopathological changes, inflammatory cytokines in serum and BALF, macrophages infiltration, inflammatory cytokine mRNA levels and inflammatory pathway (Figures 6, 7 and 8). However, pretreatment with **c26** attenuated the increase of these markers induced by LPS through inhibited the phosphorylation of ERK. In summary, the most active compound, **c26,** exhibited its anti-inflammatory activity *in vitro* and attenuated LPS-induced acute lung injury by reducing inflammatory responses *in vivo* through ERK pathway. This article provides a potential compound for the treatment of acute lung injury.

## Conclusions

In conclusion, we designed and synthesized 30 curcumin analogs based on the structure of curcumin and C66. *In vitro*, most curcumin analogs showed better activity on LPS-induced production of TNF-α and IL-6 than C66. Active compounds, **a17**, **a18**, **c9,** and **c26,** exhibited their anti-inflammatory activity in a dose-dependent manner and showed high chemical stability *in vitro*. From the perspective mechanisms, compound **c26** dose-dependently inhibited LPS-induced ERK phosphorylation in macrophages. In rat models with ALI, pretreatment with **c26** significantly attenuated LPS-induced pulmonary edema, pathological changes, inflammatory cytokines in serum and BALF, inflammatory cell infiltration, inflammatory cytokine mRNA expression and ERK phosphorylation. This presents the possibility that curcumin analogs might serve as potential agents for the treatment of ALI. Although the anti-inflammatory mechanism and underlying targets are still unknown, the beneficial effects of these compounds on LPS-induced inflammation make **c26** one of important leads in the continuing drug development and research.

## Additional files

Additional file 1: Table S1. The sequences primers. Additional file 2:
**The synthetic method of curcumin analogs and their spectral data.**
Additional file 3: Figure S1. The viability of mouse peritoneal macrophages was detected through an MTT assay after treatment active analogs for 24 h. **Figure S2.** The compounds stability was described by the curve which is consisted of absorbance at various optical density (250-600 nm) at the time 0, 0.5, 1, 2, 6, 12, and 24 h.

## References

[1] Johnson ER, Matthay MA (2010). Acute lung injury: epidemiology, pathogenesis, and treatment. J Aerosol Med Pulm Drug Deliv.

[2] Wheeler AP, Bernard GR (2007). Acute lung injury and the acute respiratory distress syndrome: a clinical review. Lancet.

[3] Levitt JE, Matthay MA (2012). Clinical review: early treatment of acute lung injury-paradigm shift toward prevention and treatment prior to respiratory failure. Crit Care.

[4] Goodman RB, Pugin J, Lee JS, Matthay MA (2003). Cytokine-mediated inflammation in acute lung injury. Cytokine Growth Factor Rev.

[5] Grommes J, Soehnlein O (2011). Contribution of neutrophils to acute lung injury. Mol Med.

[6] Patel BV, Wilson MR, O’Dea KP, Takata M (2013). TNF-induced death signaling triggers alveolar epithelial dysfunction in acute lung injury. J Immunol.

[7] Bosmann M, Grailer JJ, Zhu K, Matthay MA, Sarma JV, Zetoune FS (2012). Anti-inflammatory effects of β2 adrenergic receptor agonists in experimental acute lung injury. FASEB J.

[8] Ni Y-F, Wang J, Yan X-L, Tian F, Zhao J-B, Wang Y-J (2010). Histone deacetylase inhibitor, butyrate, attenuates lipopolysaccharide-induced acute lung injury in mice. Respir Res.

[9] Liu Z, Yang Z, Fu Y, Li F, Liang D, Zhou E (2013). Protective effect of gossypol on lipopolysaccharide-induced acute lung injury in mice. Inflamm Res.

[10] Yingkun N, Zhenyu W, Jing L, Xiuyun L, Huimin Y (2013). Stevioside protects LPS-induced acute lung injury in mice. Inflammation.

[11] Calfee CS, Matthay MA (2007). Nonventilatory treatments for acute lung injury and ARDS*. Chest J.

[12] Levitt JE, Matthay MA. Treatment of Acute Lung Injury: Historical Perspective and Potential Future Therapies. In Seminars in Respiratory and Critical Care Medicine. Copyright© 2006 by Thieme Medical Publishers, Inc., 333 Seventh Avenue, New York, NY 10001, USA.; 2006: 426–437. 10.1055/s-2006-948296 16909376

[13] O’Neal HR, Koyama T, Koehler EA, Siew E, Curtis BR, Fremont RD (2011). Prehospital statin and aspirin use and the prevalence of severe sepsis and ALI/ARDS. Crit Care Med.

[14] Ando H, Takamura T, Ota T, Nagai Y, Kobayashi K-i (2000). Cerivastatin improves survival of mice with lipopolysaccharide-induced sepsis. J Pharmacol Exp Ther.

[15] Jacobson JR, Barnard JW, Grigoryev DN, Ma S-F, Tuder RM, Garcia JG (2005). Simvastatin attenuates vascular leak and inflammation in murine inflammatory lung injury. Am J Physiol-Lung Cellular Mol Physiol.

[16] Leitman IM (2012). Curcumin for the prevention of acute lung injury in sepsis: is it more than the flavor of the month?. J Surg Res.

[17] Liang G, Shao L, Wang Y, Zhao C, Chu Y, Xiao J (2009). Exploration and synthesis of curcumin analogues with improved structural stability both *in vitro* and *in vivo* as cytotoxic agents. Bioorg Med Chem.

[18] Prasad S, Tyagi AK, Aggarwal BB (2014). Recent developments in delivery, bioavailability, absorption and metabolism of curcumin: the golden pigment from golden spice. Cancer Res Treat.

[19] Zhao C, Liu Z, Liang G (2013). Promising curcumin-based drug design: mono-carbonyl analogues of curcumin (MACs). Curr Pharm Des.

[20] Wu J, Zhang Y, Cai Y, Wang J, Weng B, Tang Q (2013). Discovery and evaluation of piperid-4-one-containing mono-carbonyl analogs of curcumin as anti-inflammatory agents. Bioorg Med Chem.

[21] Zhang Y, Zhao C, He W, Wang Z, Fang Q, Xiao B (2014). Discovery and evaluation of asymmetrical monocarbonyl analogs of curcumin as anti-inflammatory agents. Drug Design, Develop Therapy.

[22] Pan Y, Huang Y, Wang Z, Fang Q, Sun Y, Tong C (2014). Inhibition of MAPK‐mediated ACE expression by compound C66 prevents STZ‐induced diabetic nephropathy. J Cell Mol Med.

[23] Pan Y, Zhang X, Wang Y, Cai L, Ren L, Tang L (2013). Targeting JNK by a new curcumin analog to inhibit NF-kB-mediated expression of cell adhesion molecules attenuates renal macrophage infiltration and injury in diabetic mice. PLoS One.

[24] Guzel A, Kanter M, Guzel A, Yucel AF, Erboga M (2013). Protective effect of curcumin on acute lung injury induced by intestinal ischemia/reperfusion. Toxicol Ind Health.

[25] Xu F, S-h L, Yang Y-z, Guo R, Cao J, Liu Q (2013). The effect of curcumin on sepsis-induced acute lung injury in a rat model through the inhibition of the TGF-β1/SMAD3 pathway. Int Immunopharmacol.

[26] Xiao X, Yang M, Sun D, Sun S (2012). Curcumin protects against sepsis-induced acute lung injury in rats. J Surg Res.

[27] Zhong W-t, Jiang L-x, Wei J-y, Qiao A-n, Wei M-m, Soromou L-W (2013). Protective effect of esculentoside A on lipopolysaccharide-induced acute lung injury in mice. J Surg Res.

[28] Pan C, Wang J, Liu W, Liu L, Jing L, Yang Y (2012). Low tidal volume protects pulmonary vasomotor function from “second-hit” injury in acute lung injury rats. Respir Res.

[29] Wang Y-J, Pan M-H, Cheng A-L, Lin L-I, Ho Y-S, Hsieh C-Y (1997). Stability of curcumin in buffer solutions and characterization of its degradation products. J Pharm Biomed Anal.

[30] Goodman RB, Strieter RM, Martin DP, Steinberg KP, Milberg JA, Maunder RJ (1996). Inflammatory cytokines in patients with persistence of the acute respiratory distress syndrome. Am J Respir Crit Care Med.

[31] Randhawa R, Bellingan G (2007). Acute lung injury. Anaesth Intensive Care Med.

[32] Steinberg KP, Hudson LD, Goodman RB, Hough CL, Lanken PN, Hyzy R (2006). Efficacy and safety of corticosteroids for persistent acute respiratory distress syndrome. N Engl J Med.

[33] Akdis M, Burgler S, Crameri R, Eiwegger T, Fujita H, Gomez E (2011). Interleukins, from 1 to 37, and interferon-γ: receptors, functions, and roles in diseases. J Allergy Clin Immunol.

[34] Bouros D, Alexandrakis MG, Antoniou KM, Agouridakis P, Pneumatikos I, Anevlavis S (2004). The clinical significance of serum and bronchoalveolar lavage inflammatory cytokines in patients at risk for Acute Respiratory Distress Syndrome. BMC Pulm Med.

[35] van der Poll T, Keogh CV, Guirao X, Buurman WA, Kopf M, Lowry SF (1997). Interleukin-6 gene-deficient mice show impaired defense against pneumococcal pneumonia. J Infect Dis.

[36] Chen J, Wang J-B, Yu C-H, Chen L-Q, Xu P, Yu W-Y (2013). Total flavonoids of Mosla scabra leaves attenuates lipopolysaccharide-induced acute lung injury via down-regulation of inflammatory signaling in mice. J Ethnopharmacol.

[37] Liang D, Sun Y, Shen Y, Li F, Song X, Zhou E (2013). Shikonin exerts anti-inflammatory effects in a murine model of lipopolysaccharide-induced acute lung injury by inhibiting the nuclear factor-kappaB signaling pathway. Int Immunopharmacol.

[38] Wan L-M, Tan L, Wang Z-R, Liu S-X, Wang Y-L, Liang S-Y (2013). Preventive and therapeutic effects of Danhong injection on lipopolysaccharide induced acute lung injury in mice. J Ethnopharmacol.

[39] Lu Y-C, Yeh W-C, Ohashi PS (2008). LPS/TLR4 signal transduction pathway. Cytokine.

[40] Lenardo MJ, Baltimore D (1989). NF-κB: a pleiotropic mediator of inducible and tissue-specific gene control. Cell.

[41] Zhong F, Chen H, Han L, Jin Y, Wang W (2011). Curcumin attenuates lipopolysaccharide-induced renal inflammation. Biol Pharm Bull.

[42] Seger R, Krebs EG (1995). The MAPK signaling cascade. FASEB J.

[43] Kaminska B (2005). MAPK signalling pathways as molecular targets for anti-inflammatory therapy—from molecular mechanisms to therapeutic benefits. Biochimica et Biophysica Acta (BBA)-Proteins Proteomics.

[44] Kim SH, Smith CJ, Van Eldik LJ (2004). Importance of MAPK pathways for microglial pro-inflammatory cytokine IL-1β production. Neurobiol Aging.

[45] Zhan J, Liu Y, Zhang Z, Chen C, Chen K, Wang Y (2011). Effect of penehyclidine hydrochloride on expressions of MAPK in mice with CLP-induced acute lung injury. Mol Biol Rep.

[46] Fein AM, Calalang-Colucci MG (2000). Acute lung injury and acute respiratory distress syndrome in sepsis and septic shock. Crit Care Clin.

[47] Abraham E (2000). Coagulation abnormalities in acute lung injury and sepsis. Am J Respir Cell Mol Biol.

[48] Reutershan J, Basit A, Galkina EV, Ley K (2005). Sequential recruitment of neutrophils into lung and bronchoalveolar lavage fluid in LPS-induced acute lung injury. Am J Physiol-Lung Cellular Mol Physiol.

[49] Coimbra R, Melbostad H, Loomis W, Porcides RD, Wolf P, Tobar M (2006). LPS-induced acute lung injury is attenuated by phosphodiesterase inhibition: effects on proinflammatory mediators, metalloproteinases, NF-[kappa] B, and ICAM-1 expression. J Trauma Injury Infection Critical Care.

