# Supplementary material for: Anti-inflammatory effects of novel curcumin analogs in experimental acute lung injury
Source: Respir Res. 2015 Mar 24;16(1):43. doi: 10.1186/s12931-015-0199-1 (PMC4391684; doi:10.1186/s12931-015-0199-1)
Supplement: Additional file 1: Table S1. — The sequences primers. [file 12931_2015_199_MOESM1_ESM.pdf]

Additional File 1

Anti-inflammatory effects of novel curcumin analogs in experimental acute lung injury

Yali Zhang[1,2,#], Dandan Liang[1,#], Lili Dong[3], Xiangting Ge[3], Fengli Xu[3], Yuanrong Dai[3], Peng Zou[2], Shulin Yang[2,*], Guang Liang[1,*]

1. Chemical Biology Research Center at School of Pharmaceutical Sciences, Wenzhou Medical University, Wenzhou, Zhejiang 325035, China
2. School of Environmental and Biological Engineering, Nanjing University of Science and Technology, Nanjing, Jiangsu 210094, China
3. The 2nd Affiliated Hospital, Wenzhou Medical University, Wenzhou, Zhejiang 325035, China

Table S1 The sequences of primers

Primers	Sequences
Human TNF-α sense	CCCAGGGACCTCTCTCTAATC
Human TNF-α antisense	ATGGGCTACAGGCTTGTCACT
Human IL-6 sense	GCACTGGCAGAAAACAACCT
Human IL-6 antisense	TCAAACTCCAAAAGACCAGTGA
Human IL-1β sense	ACGCTCCGGGACTCACAGCA
Human IL-1β antisense	TGAGGCCCAAGGCCACAGGT
Human COX-2 sense	TTCTCCTTGAAAGGACTTATGGGTAA
Human COX-2 antisense	AGAACTTGCATTGATGGTGACTGTTT
Human β-actin sense	CCTGGCACCCAGCACAAT
Human β-actin antisense	GCCGATCCACACGGAGTACT
