# Supplementary material for: Anti-inflammatory effects of novel curcumin analogs in experimental acute lung injury
Source: Respir Res. 2015 Mar 24;16(1):43. doi: 10.1186/s12931-015-0199-1 (PMC4391684; doi:10.1186/s12931-015-0199-1)
Supplement: Additional file 2: — The synthetic method of curcumin analogs and their spectral data. [file 12931_2015_199_MOESM2_ESM.pdf]

Additional File 2

Anti-inflammatory effects of novel curcumin analogs in experimental acute lung injury

Yali Zhang[1,2,#], Dandan Liang[1,#], Lili Dong[3], Xiangting Ge[3], Fengli Xu[3], Yuanrong Dai[3], Peng Zou[2], Shulin Yang[2,*], Guang Liang[1,*]

1. Chemical Biology Research Center at School of Pharmaceutical Sciences, Wenzhou Medical University, Wenzhou, Zhejiang 325035, China

2. School of Environmental and Biological Engineering, Nanjing University of Science and Technology, Nanjing, Jiangsu 210094, China

3. The 2nd Affiliated Hospital, Wenzhou Medical University, Wenzhou, Zhejiang 325035, China

Spectral data

Synthesis of morpholine enamines 2a and 2b

To a solution of morpholine (0.12 mol) in dry toluene (70 mL) was added cyclopentanone or cyclohexanone (0.1 mol) dropwise, followed by p-toluenesulfonic acid (1.16 mmol) at room temperature. The mixture solution was then refluxed for 5h. The resulting solution was concentrated in vacuo to give morpholine enamines **2a** and **2b** with the yields of 60-85%.

Synthesis of α,β-unsaturated ketones 3a, 3b, 4a and 4b

A solution of morpholine enamines **2a** or **2b** (3.0 equiv) in Ethanol (15 mL) was added 2-trifluorobenzaldehyde or 2-nitrobenzaldehyde (1 equiv) portion wise. After refluxing for 4-5h, the resulting mixture was acidated to a pH of 5-6 by a solution of 5% HCl, diluted with brine and extracted with EtOAc (3×10 mL). The combined organic layers were washed with brine and dried over anhydrous $MgSO_4$, filtered and concentrated under reduced pressure. The residue was further purified by chromatography on silica gel to give desired ketones **3a, 3b, 4a and 4b**.

Synthesis of asymmetric MACs a1-a10, a12-a16, a19, b10-b20 and b22-b25.

To a stirring solution of **3a, 3b, 4a, 4b** (0.37 mmol) and various aromatic aldehydes (0.37 mmol) in EtOH (10 mL) was added 20% NaOH dropwise. The reaction mixture was stirred at room temperature for 10h, then quenched with saturated aqueous NH_4Cl solution (10 mL) and extracted with EtOAc (3×10 mL). The combined organic layers were washed with brine (15 mL) and dried over anhydrous $MgSO_4$, filtered, and concentrated under reduced pressure. The residue was purified by recrystallization or chromatography on silica gel to furnish target MACs **a1-a10, a12-a16, a19, b10-b20 and b22-b25**.

(2E,6E)-2-(4-morpholinobenzylidene)-6-(2-(trifluoromethyl)benzylidene)cyclohexanone **(a1)**

Red powder, 36.7% yield, m.p 176.9-179.4 ºC. ^{1}H-NMR (600 MHz, $CDCl_3$) δ (ppm) 7.921 (1H, s, H-β), 7.791(1H, s, H-β'), 7.710 (1H, d, *J* = 7.8 Hz, H-3), 7.540 (1H, t, *J* = 7.2 Hz, H-5), 7.469 (2H, d, *J* = 8.4 Hz, H-2', H-6'), 7.418 (1H, t, *J* = 7.8 Hz, H-4), 7.314 (1H, d, *J* = 7.2 Hz, H-6), 6.919 (2H, d, *J* = 8.4 Hz, H-3', H-5'), 3.872 (4H, t, *J* =

4.8 Hz, Ar-morpholine-$CH_2C\underline{H}_2OC\underline{H}_2CH_2$-),3.259 (4H, t, *J* = 4.8 Hz, Ar-morpholine-$C\underline{H}_2CH_2OCH_2C\underline{H}_2$-), 2.935 (2H, t, *J* = 5.4 Hz, Cyclohexanone-$C\underline{H}_2CH_2CH_2$-), 2.613 (2H, t, *J* = 4.8 Hz, Cyclohexanone-$CH_2CH_2C\underline{H}_2$-),1.741-1.782 (2H, m, Cyclohexan-one-$CH_2C\underline{H}_2CH_2$-). ESI-MS m/z: 428.4 $(M+H)^+$.

(2E,6E)-2-(4-(4-methylpiperazin-1-yl)benzylidene)-6-(2-(trifluoromethyl)benzylidene)cyclohexanone **(a2)**

Red powder, 50.7% yield, m.p 100.1-102.8 °C. ^{1}H-NMR (600 MHz, DMSO) δ (ppm) 7.825 (1H, s, H-β), 7.727 (2H, d, *J* = 3.6 Hz, H-2', H-6'), 7.594 (2H, d, *J* = 16.2 Hz, H-3, H-6), 7.514 (1H, t, *J* = 7.8 Hz, H-5), 7.470 (1H, s, H-β'), 7.455 (1H, t, *J* = 9 Hz, H-4), 6.995 (2H, d, *J* = 9 Hz, H-3', H-5'), 3.269 [4H, t, *J* = 4.8 Hz, Ar-N-methylpiper aine-$C\underline{H}_2CH_2N(CH_3)CH_2C\underline{H}_2$-], 2.892 (2H, t, *J* = 6 Hz, Cyclohexanone-$C\underline{H}_2CH_2CH_2$ -), 2.612 (2H, t, *J* = 6 Hz, Cyclohexanone-$CH_2CH_2C\underline{H}_2$-), 2.434 [4H, t, *J* = 4.8 Hz, Ar-N-methylpiperaine-$CH_2C\underline{H}_2N(CH_3)C\underline{H}_2CH_2$-], 2.217(3H, s, Ar-N-methylpiperaine -CH_3), 1.625-1.795 (2H, m, Cyclohexanone-$CH_2C\underline{H}_2CH_2$-). ESI-MS m/z: 441.6 $(M+H)^+$.

(2E,6E)-2-(4-(3-(dimethylamino)propoxy)benzylidene)-6-(2-(trifluoromethyl)benzyli dene)cyclohexanone **(a3)**

Yellow oil, 51.6% yield. ^{1}H-NMR (600 MHz, $CDCl_3$) δ (ppm) 7.894 (1H, s, H-β), 7.825 (1H, s, H-β'), 7.647 (2H, d, *J* = 3.6 Hz, H-2', H-6'), 7.572 (1H, d, *J* = 9 Hz, H-3), 7.564 (1H, d, *J* = 9 Hz, H-5), 7.440 (1H, d, *J* = 7.8 Hz, H-6), 7.344 (1H, t, *J* = 5.4 Hz, H-4), 7.034 (2H, d, *J* = 5.4 Hz, H-3', H-5'), 4.053 [2H, t, *J* = 6.6 Hz, Ar-$OC\underline{H}_2CH_2CH_2N(CH_3)_2$], 2.503 (2H, t, *J* = 1.8 Hz, Cyclohexanone-$C\underline{H}_2CH_2CH_2$-), 2.497 (2H, t, *J* = 1.8 Hz, Cyclohexanone-$CH_2CH_2C\underline{H}_2$-), 2.364 [2H, t, *J* = 3.6 Hz, Ar-$OCH_2CH_2C\underline{H}_2N(CH_3)_2$], 2.155 [6H, s, Ar-$OCH_2CH_2CH_2N(C\underline{H}_3)_2$], 1.825-1.862 [2H, m, Ar-$OCH_2C\underline{H}_2CH_2N(CH_3)_2$], 1.255-1.412 (2H, m, Cyclohexanone-$CH_2C\underline{H}_2$ CH_2-). ESI-MS m/z: 444.4 $(M+H)^+$.

(2E,6E)-2-(4-(piperidin-1-yl)benzylidene)-6-(2-(trifluoromethyl)benzylidene)cyclohe

xanone **(a4)**

Red oil, 31.7% yield. ^{1}H-NMR (600 MHz, $CDCl_3$) δ (ppm) 7.705 (1H, d, *J* = 7.8 Hz, H-2'), 7.633 (1H, d, *J* = 7.8 Hz, H-6'), 7.535 (1H, t, *J* = 7.8 Hz, H-5), 7.478 (1H, s, H-β), 7.440 (1H, d, *J* = 9 Hz, H-3), 7.352 (1H, t, *J* = 9 Hz, H-4), 7.351 (1H, s, H-β'), 7.313 (1H, d, *J* = 7.8 Hz, H-6), 6.905 (1H, d, *J* = 9Hz, H-3'), 6.868 (1H, d, *J* = 8.4 Hz, H-5'), 3.285 (4H, t, *J* = 10.2 Hz, Ar-tetrahydropyridine-$C\underline{H}_2CH_2CH_2CH_2C\underline{H}_2$-), 2.754 (2H, t, *J* = 5.4 Hz, Cyclohexanone-$C\underline{H}_2CH_2CH_2$), 2.604 (2H, t, *J* = 4.8 Hz, Cyclohexanone-$CH_2CH_2C\underline{H}_2$-), 1.738-1.780 (2H, m, Cyclohexanone-$CH_2C\underline{H}_2CH_2$-), 1.678-1.693 (2H, m, Ar-tetrahydropyridine-$CH_2CH_2C\underline{H}_2CH_2CH_2$-), 1.600-1.632 (4H, m, Ar-tetrahydropyridine-$CH_2C\underline{H}_2CH_2C\underline{H}_2CH_2$-). ESI-MS m/z: 426.2 $(M+H)^+$.

(2E,6E)-2-(2-bromo-5-fluorobenzylidene)-6-(2-(trifluoromethyl)benzylidene)cyclohexanone **(a5)**

Yellow oil, 54.6% yield. ^{1}H-NMR (600 MHz, $CDCl_3$) δ (ppm) 7.962 (1H, s, H-β), 7.850 (1H, s, H-β'), 7.725 (1H, d, *J* = 7.8 Hz, H-3), 7.598 (1H, d, *J* = 5.4 Hz, H-3'), 7.557 (1H, d, *J* = 7.8 Hz, H-5), 7.441 (1H, t, *J* =7.8 Hz, H-4), 7.330 (1H, d, *J* = 7.8 Hz, H-6), 7.041 (1H, d, *J* = 6.6 Hz, H-4'), 6.22-6.941 (1H, m, H-6'), 2.739 (2H, t, *J* = 4.8Hz, Cyclohexanone-$C\underline{H}_2CH_2CH_2$-), 2.647 (2H, t, *J* = 5.4 Hz, Cyclohexanone-CH_2 $CH_2C\underline{H}_2$-), 1.739-1.770 (2H, m, Cyclohexanone-$CH_2C\underline{H}_2CH_2$-). ESI-MS m/z: 439.6 $(M)^+$.

(2E,6E)-2-(2-bromo-6-fluorobenzylidene)-6-(2-(trifluoromethyl)benzylidene)cyclohexanone **(a6)**

Yellow powder, 44.7% yield, m.p 99.2-102.5 °C. ^{1}H-NMR (600 MHz, $CDCl_3$) δ (ppm) 7.968 (1H, s, H-β), 7.929 (1H, s, H-β'), 7.724 (1H, d, *J* = 7.8 Hz, H-3), 7.542 (1H, d, *J* = 6 Hz, H-5), 7.532 (1H, t, *J* = 8.4 Hz, H-4), 7.507 (2H, d, *J* = 12 Hz, H-6, H-3'), 7.443 (1H, d *J* = 7.8 Hz, H-5'), 7.416 (1H, t, *J* = 5.4 Hz, H-4'), 2.647 (4H, t, *J* = 6 Hz, Cyclohexanone-$C\underline{H}_2CH_2C\underline{H}_2$-), 1.709-1.729 (2H, m, Cyclohexanone-$CH_2C\underline{H}_2$ CH_2-). ESI-MS m/z: 439.4 $(M)^+$.

(2E,6E)-2-(4-bromobenzylidene)-6-(2-(trifluoromethyl)benzylidene)cyclohexanone **(a7)**

Yellow powder, 25.6% yield, m.p 129.7-132.3 ^oC. ^1H-NMR (600 MHz, $CDCl_3$) δ (ppm) 7.945 (1H, s, H-β), 7.720 (2H, d, *J* = 9.0 Hz , H-2', H-6'), 7.557 (1H , d, *J* = 7.8 Hz, H-3), 7.537 (2H, d, *J* = 6.4 Hz, H-3', H-5'), 7.434 (1H, t, *J* = 7.2 Hz, H-5), 7.331 (1H, s, H-β'), 7.327 (1H, d, *J* = 6.0 Hz, H-6), 7.317 (1H, t, *J* = 4.2 Hz, H-4), 2.874 (2H, t, *J* = 5.4 Hz, Cyclohexanone-C$\underline{H}_2$$CH_2$$CH_2$-), 2.631 (2H, t, *J* = 5.4 Hz, Cyclohexanone -$CH_2$$CH_2C\underline{H}_2$-), 1.743-1.763 (2H, m, Cyclohexanone-CH_2C$\underline{H}_2$$CH_2$-). ESI-MS m/z: 423.1 $(M)^+$.

(2E,6E)-2-(2,5-dibromobenzylidene)-6-(2-(trifluoromethyl)benzylidene)cyclohexanone **(a8)**

Yellow oil, 54.6% yield. ^1H-NMR (600 MHz, $CDCl_3$) δ(ppm) 7.959 (1H, s, H-β), 7.741 (1H, s, H-β'), 7.726 (1H, d, *J* = 7.8 Hz, H-3), 7.599 (1H, t, *J* = 7.2 Hz, H-5), 7.500 (1H, d, *J* = 8.4 Hz, H-3'), 7.448 (1H, d, *J* = 7.2 Hz, H-4'), 7.427 (2H, *t*, *J* = 2.4 Hz, H-4, H-6'), 7.332 (1H, d, *J* = 5.4 Hz, H-6), 2.732 (2H, t, *J* = 4.8 Hz, Cyclohexanone-C$\underline{H}_2$$CH_2$$CH_2$-), 2.648 (2H, t, *J* = 5.4 Hz, Cyclohexanone-$CH_2$$CH_2C\underline{H}_2$-),1.741 -1.761 (2H, m, Cyclohexanone-CH_2C$\underline{H}_2$$CH_2$-). ESI-MS m/z: 501.3 $(M+H)^+$.

(2E,6E)-2-(3,4-dimethoxybenzylidene)-6-(2-nitrobenzylidene)cyclohexano **(a9)**

Yellow powder, 70.6% yield, m.p 131.8-135.5 ^oC. ^1H-NMR (600 MHz, $CDCl_3$) δ(ppm) 8.225 (1H, d, *J* = 7.2 Hz, H-3), 7.947 (1H, s, H-β), 7.792 (1H, s, H-β'), 7.640 (1H, t, *J* = 15.0 Hz, H-4), 7.500 (1H, t, *J* = 15.6 Hz, H-5), 7.370 (1H, d, *J* = 7.8 Hz, H-6), 7.123 (1H, d, *J* = 8,4 Hz, H-6'), 7.027 (1H, s, H-2'), 6.915 (1H, d, *J* = 8.4 Hz, H-5'), 3.928 (3H, s, 3'-OCH_3), 3.914 (3H, s, 4'-OCH_3), 2.949 (2H, t, *J* = 10.2 Hz, Cyclohexanone-C$\underline{H}_2$$CH_2$$CH_2$-), 2.605 (2H, t, *J* = 10.2 Hz, Cyclohexanone-$CH_2$$CH_2$ C$\underline{H}_2$-), 1.766-1.808 (2H, m, Cyclohexanone-CH_2C$\underline{H}_2$$CH_2$-). ESI-MS m/z: 380.1 $(M+H)^+$.

(2E,6E)-2-(2,3-dimethoxybenzylidene)-6-(2-nitrobenzylidene)cyclohexano **(a10)**
Orange powder, 63.2% yield, m.p 124.3-129.1 $^{\circ}$C. ^{1}H-NMR (600 MHz, $CDCl_3$) δ(ppm) 8.127 (1H, d, *J* = 8.4 Hz, H-3), 7.955 (2H, s, H-β, H-β'), 7.640 (1H, t, *J* = 14.4 Hz, H-4), 7.501 (1H, t, *J* = 14.4 Hz, H-5), 7.376 (1H, d, *J* = 7.8 Hz, H-6), 7.065 (1H, t, *J* = 15.0 Hz, H-5'), 6.930 (2H, d, *J* = 8.4 Hz, H-4', H-6'), 3.889 (3H, s, 2'-OCH_3), 3.834 (3H, s, 3'-OCH_3), 2.804 (2H, t, *J* = 10.2 Hz, Cyclohexanone-$C\underline{H}_2CH_2$ CH_2-), 2.604 (2H, t, *J* = 10.2 Hz, Cyclohexanone-$CH_2CH_2C\underline{H}_2$-), 1.709-1.750 (2H, m, Cyclohexanone-$CH_2C\underline{H}_2CH_2$-). ESI-MS m/z: 380.0 $(M+H)^+$.

(2E,6E)-2-(2-fluoro-4-methoxybenzylidene)-6-(2-nitrobenzylidene)cyclohexanone **(a12)**

Yellow oil, 40.7% yield, ^{1}H-NMR (600 MHz, $CDCl_3$) δ(ppm) 8.125 (1H, d, *J* = 8.4 Hz, H-3), 7.947 (1H, s, H-β), 7.850 (1H, s, H-β'), 7.640 (1H, t, *J* = 14.4 Hz, H-4), 7.500 (1H, t, *J* = 15.6 Hz, H-5), 7.368 (1H, d, *J* = 8.4 Hz, H-6), 7.345 (1H, s, H-3'), 6.727 (1H, d, *J* = 10.8 Hz, H-6'), 6.675 (1H, d, *J* = 14.4 Hz, H-5'), 3.836 (3H, s, 4'-OCH_3), 2.815 (2H, t, *J* = 10.8 Hz, Cyclohexanone-$C\underline{H}_2CH_2CH_2$-), 2.604 (2H, t, *J* = 10.2 Hz, Cyclohexanone-$CH_2CH_2C\underline{H}_2$-), 1.738-1.780 (2H, m, Cyclohexanone -$CH_2C\underline{H}_2CH_2$-). ESI-MS m/z: 368.0 $(M+H)^+$.

(2E,6E)-2-(2-methoxybenzylidene)-6-(2-nitrobenzylidene)cyclohexanone **(a13)**
Yellow powder, 69.4% yield, m.p 148.8-153.9 $^{\circ}$C. ^{1}H-NMR (600 MHz, $CDCl_3$) δ(ppm) 8.119 (1H, d, *J* = 8.4 Hz, H-3), 8.030 (1H, s, H-β), 7.943 (1H, s, H-β'), 7.635 (1H, t, *J* = 14.4 Hz, H-4), 7.495 (1H, t, *J* = 15.6 Hz, H-5), 7.368 (1H, d, *J* = 7.8 Hz, H-6), 7.336 (1H, t, *J* = 15.6 Hz, H-5'), 7.317 (1H, d, *J* = 13.8 Hz, H-6'), 6.967 (1H, t, *J* = 14.4 Hz, H-4'), 6.928 (1H, d, *J* = 8.4 Hz, H-3'), 3.875 (3H, s, 2'-OCH_3), 2.847 (2H, t, *J* = 10.8 Hz, Cyclohexanone-$C\underline{H}_2CH_2CH_2$-), 2.607 (2H, t, *J* = 10.8 Hz, Cyclohexan one-$CH_2CH_2C\underline{H}_2$-), 1.720-1.761 (2H, m, Cyclohexanone-$CH_2C\underline{H}_2CH_2$-). ESI-MS m/z: 350.1 $(M+H)^+$.

(2E,6E)-2-(3-bromo-4-methoxybenzylidene)-6-(2-nitrobenzylidene)cyclohexanone

(a14)

Yellow powder, 66.0% yield, m.p: 183.2-187.2 ^{o}C. ^{1}H-NMR (600 MHz, $CDCl_3$) δ(ppm) 8.130 (1H, d, *J* = 7.8 Hz, H-3), 7.948 (1H, s, H-2'), 7.712 (2H, s, H-β, H-β'), 7.644 (1H, t, *J* = 15.0 Hz, H-4), 7.505 (1H, t, *J* = 15.0 Hz, H-5), 7.419 (1H, d, *J* = 10.2 Hz, H-6), 7.368 (1H, d, *J* = 7.2 Hz, H-6'), 6.940 (1H, d, *J* = 8.4 Hz, H-5'), 3.942 (3H, s, 4'-OCH_3), 2.910 (2H, t, *J* = 10.2 Hz, Cyclohexanone-$C\underline{H}_2CH_2CH_2$-), 2.604 (2H, t, *J* = 10.2 Hz, Cyclohexanone-$CH_2CH_2C\underline{H}_2$-), 1.765-1.807 (2H, m, Cyclohexanone-$CH_2C\underline{H}_2CH_2$-). ESI-MS m/z: 429.8 $(M+H)^+$.

(2E,6E)-2-(5-bromo-2-ethoxybenzylidene)-6-(2-nitrobenzylidene)cyclohexanone

(a15)

Yellow powder, 67.8% yield, m.p 154.1-158.6 ^{o}C. ^{1}H-NMR (600 MHz, $CDCl_3$) δ(ppm) 8.127 (1H, d, *J* = 8.4 Hz, H-3), 7.950 (1H, s, H-β), 7.924 (1H, s, H-β'), 7.643 (1H, t, *J* = 15.0 Hz, H-4), 7.504 (1H, t, *J* = 14.4 Hz, H-5), 7.405 (1H, s, H-6'), 7.374 (2H, d, *J* = 7.8 Hz, H-6, H-3'), 6.787 (1H, d, *J* = 8.4 Hz, H-4'), 4.048-4.083 (2H, m, Ar-$OC\underline{H}_2CH_3$), 2.832 (2H, t, *J* = 12.0 Hz, Cyclohexanone-$C\underline{H}_2CH_2CH_2$-), 2.615 (2H, t, *J* = 12.0 Hz, Cyclohexanone-$CH_2CH_2C\underline{H}_2$-), 1.740-1.781 (2H, m, Cyclohexanone -$CH_2C\underline{H}_2CH_2$-), 1.453 (3H, t, *J* = 13.8 Hz, Ar-$OCH_2C\underline{H}_3$). ESI-MS m/z: 443.8 $(M+H)^+$.

(2E,6E)-2-(2-fluoro-5-methoxybenzylidene)-6-(2-nitrobenzylidene)cyclohexanone

(a16)

Yellow powder, 77.8% yield, m.p 139.3-144.0 ^{o}C. ^{1}H-NMR (600 MHz, $CDCl_3$) δ(ppm) 8.133 (1H, d, *J* = 7.8 Hz, H-3), 7.957 (1H, s, H-β), 7.792 (1H, s, H-β'), 7.647 (1H, t, *J* =15.0 Hz, H-4), 7.510 (1H, t, *J* = 15.6 Hz, H-5), 7.373 (1H, d, *J* = 7.8 Hz, H-6), 7.032 (1H, t, *J* = 18 Hz, H-3'), 6.840-6.852 (2H, m, H-4', H-6'), 3.803 (3H, s, 5'-OCH_3), 2.800 (2H, t, *J* = 11.4 Hz, Cyclohexanone-$C\underline{H}_2CH_2CH_2$-), 2.615 (2H, t, *J* = 10.2 Hz, Cyclohexanone-$CH_2CH_2C\underline{H}_2$-), 1.735-1.777 (2H, m, Cyclohexanone-$CH_2C\underline{H}_2CH_2$-). ESI-MS m/z: 367.9 $(M+H)^+$.

(2E,6E)-2-(4-ethoxybenzylidene)-6-(2-nitrobenzylidene)cyclohexanone **(a19)**
Yellow powder, 67.0% yield, m.p 142.3-143.7 ºC. ^{1}H-NMR (600 MHz, DMSO) δ(ppm) 8.158 (1H, d, *J* = 7.2 Hz, H-3), 7.803 (1H, t, *J* = 13.8 Hz, H-4), 7.756 (1H, s, H-β), 7.652 (1H, t, *J* = 13.8 Hz, H-5), 7.637 (1H, s, H-β'), 7.570 (1H, d, *J* = 7.2 Hz, H-6), 7.540 (2H, d, *J* = 9.0 Hz, H-2', H-6'), 7.014 (2H, d, *J* = 9.0 Hz, H-3', H-5'), 4.071-4.106 (2H, m, 4'-$OC\underline{H}_2CH_3$), 2.896 (2H, t, *J* = 10.8 Hz, Cyclohexanone-$C\underline{H}_2CH_2CH_2$-), 2.619 (2H, t, *J* = 10.8 Hz, Cyclohexanone-$CH_2CH_2C\underline{H}_2$-), 1.674-1.715 (2H, m, Cyclohexanone-$CH_2C\underline{H}_2CH_2$-), 1.345 (3H, t, *J* = 13.8 Hz, 4'-$OCH_2C\underline{H}_3$). ESI-MS m/z: 364.9 $(M+H)^{+}$.

(2E,5E)-2-(2,3-dimethoxybenzylidene)-5-(2-nitrobenzylidene)cyclopentanone **(b10)**
Yellow powder, 60.9% yield, m.p 120.8-124.0 ºC. ^{1}H-NMR (600 MHz, $CDCl_3$) δ(ppm) 8.072 (1H, d, *J* = 8.4 Hz, H-3), 7.961 (1H, s, H-β), 7.821 (1H, s, H-β'), 7.656 (1H, t, *J* = 14.4 Hz, H-4), 7.573 (1H, d, *J* = 7.2 Hz, H-6), 7.515 (1H, t, *J* = 15.0 Hz, H-5), 7.132 (1H, d, *J* = 7.2 Hz, H-6'), 7.098 (1H, t, *J* = 15.6 Hz, H-5'), 6.972 (1H, d, *J* = 7.8 Hz, H-4'), 3.896 (3H, s, 2'-OCH_3), 3.877 (3H, s, 3'-OCH_3), 3.003 (2H, t, *J* = 7.8 Hz, Cyclopentanone-$C\underline{H}_2CH_2$-), 2.890 (2H, t, *J* = 7.8 Hz, Cyclopentanone-$CH_2C\underline{H}_2$-). ESI-MS m/z: 366.1 $(M+H)^{+}$.

(2E,5E)-2-(3-bromo-4-methoxybenzylidene)-5-(2-(trifluoromethyl)benzylidene)cyclopentanone **(b14)**
Yellow powder, 61.9% yield, m.p 95.4-100.8 ºC. ^{1}H-NMR (600 MHz, $CDCl_3$) δ (ppm) 7.871 (1H, s, H-2'), 7.813 (1H, s, H-β), 7.748 (1H, d, *J* = 7.8 Hz, H-3), 7. 585 (1H, d, *J* = 7.2 Hz, H-6'), 7.579 (1H, t, *J* = 7.2 Hz, H-5), 7.546 (1H, d, *J* = 8.4 Hz, H-6), 7.470 (1H, t, *J* = 7.2 Hz, H-4), 7.050(1H, s, H-β'), 6.799 (1H, d, *J* = 4.8 Hz, H-5'), 2.969 (2H, t, *J* = 4.2 Hz, Cyclopentanone-$C\underline{H}_2CH_2$-), 2.948 (2H, t, *J* = 4.2 Hz, Cyclopentanone-$CH_2C\underline{H}_2$-). ESI-MS m/z: 439.2 $(M)^{+}$.

(2E,5E)-2-(4-ethoxybenzylidene)-5-(2-(trifluoromethyl)benzylidene)cyclopentanone **(b19)**
Yellow oil, 42.4% yield. ^{1}H-NMR (600 MHz, $CDCl_3$) δ (ppm) 7.827 (1H, s, H-β),

7.738 (1H, d, *J* = 7.8 Hz, H-3), 7. 650 (1H, d, *J* = 1.8 Hz, H-6), 7.601 (1H, t, *J* = 7.8 Hz, H-5), 7.583 (1H, s, H-β'), 7.550 (2H, d, *J* = 9 Hz, H-2', H-6'), 7.447 (1H, t, *J* = 7.8 Hz, H-4), 6.951 (2H, d, *J* = 9 Hz, H-3', H-5'), 4.072-4.107 (2H, m, 4'-O$C\underline{H}_2CH_3$), 3.043 (2H, t, *J* = 4.2 Hz, Cyclopentanone-$C\underline{H}_2CH_2$-), 2.970 (2H, t, *J* = 4.2 Hz, Cyclopentanone-$CH_2C\underline{H}_2$-), 1.442 (3H, t, *J* = 7.2 Hz, 4'-O$CH_2C\underline{H}_3$). ESI-MS m/z: 373.2 $(M+H)^+$.

(2E,5E)-2-(2-(trifluoromethyl)benzylidene)-5-(2,4,6-trimethylbenzylidene)cyclopentanone **(b20)**

Yellow powder, 65.4% yield, m.p 135.6-138.5 °C. ^{1}H-NMR (600 MHz, $CDCl_3$) δ (ppm) 7.849 (1H, t, *J* = 2.4 Hz, H-5), 7.743 (1H, d, *J* = 7.8 Hz, H-3), 7.671 (1H, s, H-β), 7.569 (1H, s, H-β'), 7.572 (1H, d, *J* = 3.6 Hz, H-6), 7.452 (1H, t, *J* = 4.2 Hz, H-4), 6.899 (2H, s, H-3',H-5'), 2.853 (2H, t, *J* = 7.2 Hz, Cyclopentanone-$C\underline{H}_2CH_2$-), 2.470 (2H, t, *J* = 7.2 Hz, Cyclopentanone-$CH_2C\underline{H}_2$-), 2.299 (3H, s, 4'-CH_3), 2.189 (6H, s, 2'-CH_3, 6'-CH_3). ESI-MS m/z: 371.5 $(M+H)^+$.

(2E,5E)-2-(2-bromo-4,5-dimethoxybenzylidene)-5-(2-(trifluoromethyl)benzylidene)cyclopentanone **(b22)**

Yellow powder, 45.8% yield, m.p 177.8-179.1 °C. ^{1}H-NMR (600 MHz, $CDCl_3$) δ (ppm) 7.853 (2H, s, H-β, H-β'), 7.745 (1H, d, *J* = 7.8 Hz, H-3), 7. 578 (1H, t, *J* = 6.6 Hz, H-5), 7.568 (1H, d, *J* = 6.6 Hz, H-6), 7.460 (1H, t, *J* = 6 Hz, H-4), 7.138 (1H, s, H-3'), 7.075 (1H, s, H-6'), 3.922 (3H, s, 5'-OCH_3), 3.888 (3H, s, 4'-OCH_3), 2.996 (2H, t, *J* = 3.6 Hz, Cyclopentanone-$C\underline{H}_2CH_2$-), 2.961 (2H, t, *J* = 3.6 Hz, Cyclopentanone-$CH_2C\underline{H}_2$-). ESI-MS m/z: 469.0 $(M)^+$.

(2E,5E)-2-((5-methylthiophen-2-yl)methylene)-5-(2-(trifluoromethyl)benzylidene)cyclopentanone **(b23)**

Yellow oil, 66.7% yield. ^{1}H-NMR (600 MHz, $CDCl_3$) δ (ppm) 7.799 (1H, s, H-β), 7.731 (1H, d, *J* = 7.8 Hz, H-3), 7.604 (1H, s, H-β'), 7.610 (1H, d, *J* = 7.2 Hz, H-3'), 7.585 (1H, t, *J* = 7.2 Hz, H-5), 7.446 (1H, t, *J* = 3.6 Hz, H-4), 7.234 (1H, d, *J* = 3.6 Hz,

H-6), 6.841 (1H, d, *J* = 3 Hz, H-4'), 2.989 (2H, t, *J* = 6 Hz, Cyclopentanone-$C\underline{H}_2CH_2$-), 2.927 (2H, t, *J* = 6 Hz, Cyclopentanone-$CH_2C\underline{H}_2$-), 2.563 (3H,s, thiophen-CH_3). ESI-MS m/z: 349.2 $(M+H)^+$.

(2E,5E)-2-((3-methylthiophen-2-yl)methylene)-5-(2-(trifluoromethyl)benzylidene)cyclopentanone **(b24)**

Yellow powder, 65.4% yield, m.p 139.7-142.7 ^oC. ^1H-NMR (600 MHz, $CDCl_3$) δ (ppm) 7.932 (1H, s, H-β), 7.818 (1H, s, H-β'), 7.739 (1H, d, *J* = 8.4 Hz, H-5'), 7.618 (1H, d, *J* = 7.8 Hz, H-3), 7.590 (1H, t, *J* = 7.8 Hz, H-5), 7.488 (1H, d, *J* = 5.4 Hz, H-4'), 7.450 (1H, t, *J* = 7.2 Hz, H-4), 7.005 (1H, d, *J* = 5.4 Hz, H-6) 3.001 (2H, t, *J* = 3.6 Hz, Cyclopentanone-$C\underline{H}_2CH_2$-), 2.969 (2H, t, *J* = 3.6 Hz, Cyclopentanone-$CH_2C\underline{H}_2$-), 2.462 (3H, s, thiophen-CH_3). ESI-MS m/z: 349.8 $(M+H)^+$.

(2E,5E)-2-((5-bromo-1H-indol-3-yl)methylene)-5-(2-(trifluoromethyl)benzylidene)cyclopentanone **(b25)**

Red powder, 35.4% yield, m.p 113.2-116.8 ^oC. ^1H-NMR (600 MHz, DMSO) δ (ppm) 12.189 (1H, s, -NH), 8.077 (1H, s, H-2'), 7.948 (1H, s, H-β), 7.897 (1H, d, *J* = 2.4 Hz, H-3), 7.763 (1H, t, *J* = 7.2 Hz, H-4), 7.701 (1H, d, *J* = 8.4 Hz, H-6), 7.596 (1H, s, H-β'), 7.587 (1H, s, H-4'), 7.575(1H, d, *J* =3.6 Hz, H-6'), 7.547 (1H, d, *J* = 2.4 Hz, H-7'), 7.440 (1H, t, *J* = 8.4 Hz, H-5), 3.047 (2H, t, *J* = 6.6 Hz, Cyclopentanone-$C\underline{H}_2CH_2$-), 2.912 (2H, t, *J* = 6.6 Hz, Cyclopentanone-$CH_2C\underline{H}_2$-). ESI-MS m/z: 446.1 $(M)^+$.

General procedure for synthesis of a11, a17,a18 and b21

To a solution of enamine **2a** or **2b** (0.40 mmol) and corresponding arylaldehyde (0.40 mmol) in EtOH (5 mL) was catalyzed by dry hydrogen chloride at room temperature. After 24 h, the resulting solution was diluted with saturated aqueous $NaHCO_3$, the EtOH was then removed under reduced pressure and extracted with EtOAc (3×10 mL). The combined organic layers were washed with brine, dried over anhydrous $MgSO_4$, filtered, and concentrated under reduced pressure. The residue

was further purified by flash column chromatography to provide MACs analogs **a11, a17, a18 and b21.**

(2E,6E)-2-(2-hydroxy-3-methoxybenzylidene)-6-(2-nitrobenzylidene)cyclohexanone **(a11)**

Yellow powder, 65.7% yield, m.p 151.0-154.1 °C. ^{1}H-NMR (600 MHz, $CDCl_3$) δ(ppm) 8.117 (1H, t, *J* = 16.8 Hz, H-4), 8.005 (1H, d, *J* = 7.8 Hz, H-3), 7.700 (1H, s, H-β), 7.669 (1H, s, H-β'), 7.598 (1H, t, *J* = 15.0 Hz, H-5), 7.556 (1H, d, *J* = 7.2 Hz, H-6), 7.482 (1H, t, *J* = 15.6 Hz, H-5'), 6.943 (1H, d, *J* = 7.8 Hz, H-6'), 6.768 (1H, d, *J* = 7.8 Hz, H-4'), 4.495 (1H, s, Ar-OH), 3.898 (3H, s, 3'-OCH_3), 2.671 (2H, t, *J* = 15.8 Hz, Cyclohexanone-$C\underline{H}_2CH_2CH_2$-), 2.557 (2H, t, *J* = 15.8 Hz, Cyclohexanone-CH_2 $CH_2C\underline{H}_2$-), 1.789-1.837 (2H, m, Cyclohexanone-$CH_2C\underline{H}_2CH_2$-). ESI-MS m/z: 364.9 M^+.

(2E,6E)-2-(4-hydroxy-3-methoxybenzylidene)-6-(2-nitrobenzylidene)cyclohexanone **(a17)**

Orange powder, 50.6% yield, m.p 165.4-167.1 °C. ^{1}H-NMR (600 MHz, DMSO) δ(ppm) 9.608 (1H, s, Ar-OH), 8.154 (1H, d, *J* = 8.4 Hz, H-3), 7.800 (1H, t, *J* = 15.0 Hz, H-4), 7.748 (1H, s, H-β), 7.649 (1H, t, *J* = 15.6 Hz, H-5), 7.7624 (1H, s, H-β'), 7.568 (1H, d, *J* = 7.8 Hz, H-6), 7.153 (1H, s, H-2'), 7.078 (1H, d, *J* = 10.2 Hz, H-5'), 6.868 (1H, d, *J* = 8.4 Hz, H-6'), 3.816 (3H, s, Ar-OCH_3), 2.918 (2H, t, *J* = 10.2 Hz, Cyclohexanone-$C\underline{H}_2CH_2CH_2$-), 2.612 (2H, t, *J* = 10.8 Hz, Cyclohexanone-CH_2CH_2 $C\underline{H}_2$-), 1.678-1.719 (2H, m, Cyclohexanone-$CH_2C\underline{H}_2CH_2$-). ESI-MS m/z: 365.9 $(M)^+$.

(2E,6E)-2-(3-hydroxy-4-methoxybenzylidene)-6-(2-nitrobenzylidene)cyclohexanone **(a18)**

Yellow powder, 47.0% yield, m.p 166.4-170.8 °C. ^{1}H-NMR (600 MHz, $CDCl_3$) δ(ppm) 8.122 (1H, d, *J* = 8.4 Hz, H-3), 7.940 (1H, s, H-β), 7.750 (1H, s, H-β'), 7.638 (1H, t, *J* = 15.0 Hz, H-4), 7.490 (1H, t, *J* = 17.4 Hz, H-5), 7.367 (1H, d, *J* = 7.8 Hz, H-6), 7.123 (1H, s, H-2'), 7.033 (1H, d, *J* = 15.0 Hz, H-6'), 6.888 (1H, d, *J* = 8.4 Hz,

H-5'), 5.534 (1H, s, 3'-OH), 3.939 (3H, s, 4'-OCH_3), 2.932 (2H, t, *J* = 10.8 Hz, Cyclohexanone-$C\underline{H}_2CH_2CH_2$-), 2.595 (2H, t, *J* = 10.8 Hz, Cyclohexanone-$CH_2CH_2C\underline{H}_2$-), 1.750-1.791 (2H, m, Cyclohexanone-$CH_2C\underline{H}_2CH_2$-). ESI-MS m/z: 364.9 (M^+).

(2E,5E)-2-(3-bromo-4-hydroxybenzylidene)-5-(2-(trifluoromethyl)benzylidene)cyclopentanone **(b21)**

Yellow powder, 53.2% yield, m.p 164.2-166.9 °C. ^{1}H-NMR (600 MHz, $CDCl_3$) δ (ppm) 7.845 (1H, s, H-2'), 7.747 (1H, d, *J* = 7.8 Hz, H-3), 7.666 (1H, d, *J* = 7.8 Hz, H-6'), 7.601 (1H, s, H-β), 7. 598 (1H, d, *J* = 4.2 Hz, H-6), 7.504 (1H, s, H-β'), 7.474 (1H, t, *J* = 6 Hz, H-5), 7.421 (1H, t, *J* = 6 Hz, H-4), 7.086 (1H, d, *J* = 8.4 Hz, H-5'), 3.033 (2H, t, *J* = 6 Hz, Cyclopentanone-$C\underline{H}_2CH_2$-), 2.979 (2H, t, *J* = 6 Hz, Cyclopentanone-$CH_2C\underline{H}_2$-). ESI-MS m/z: 422.9 $(M)^+$.

Synthesis of α,β-unsaturated ketones 5a, 5b

To a solution of 3,4-dimethoxybenzaldehyde or 2-methoxybenzaldehyde (1.00 equiv) in acetone (15 mL) was added 20% NaOH dropwise (1 mL). After stirring at room temperature for 4 h, the resulting mixture was diluted with brine and extracted with EtOAc (3×10 mL). The combined organic layers were washed with brine and dried over anhydrous $MgSO_4$, filtered and concentrated under reduced pressure. The residue was further purified by chromatography on silica gel to give desired ketones **5a, 5b**.

General procedure for synthesis of c9, c26

To a solution of 3,4-dimethoxybenzaldehyde or 2-methoxybenzaldehyde (1.0 equiv) and 2-nitrobenzaldehyde (1.0 equiv) in dioxane suspension (15 mL), which NaOH power was added in dioxane, was added 20% NaOH dropwise (0.5 mL). After stirring at room temperature for 5 h, the resulting mixture was diluted with brine and extracted with EtOAc (3×10 mL). The combined organic layers were washed with brine and dried over anhydrous $MgSO_4$, filtered and concentrated under reduced pressure. The residue was further purified by chromatography on silica gel to afford target compounds **c9, c26**.

(1E,4E)-1-(3,4-dimethoxyphenyl)-5-(2-nitrophenyl)penta-1,4-dien-3-one **(c9)**

Yellow powder, 70.0% yield, m.p 115.4-120.1 ºC. ^{1}H-NMR (600 MHz, $CDCl_3$) δ(ppm) 8.106 (1H, d, *J* = 16.2 Hz, H-β), 8.073 (1H, d, *J* = 8.4 Hz, H-3), 7.733 (1H, d, *J* = 16.2 Hz, H-β'), 7.725 (1H, d, *J* = 6.6 Hz, H-6), 7.678 (1H, t, *J* = 15.0 Hz, H-4), 7.562 (1H, t, *J* = 15.0 Hz, H-5), 7.225 (1H, d, *J* = 10.2 Hz, H-6'), 7.147 (1H, s, H-2'), 6.978 (1H, d, *J* = 16.2 Hz, H-α), 6.930 (1H, d, *J* = 15.6 Hz, H-α'), 6.906 (1H, d, *J* = 6.6 Hz, H-5'), 3.950 (3H, s, 3'-OCH_3), 3.938 (3H, s, 4'-OCH_3). ESI-MS m/z: 340.1 $(M+H)^+$.

(1E,4E)-1-(2-methoxyphenyl)-5-(2-nitrophenyl)penta-1,4-dien-3-one **(c26)**

Yellow powder, 60.1% yield, m.p 141.9-144.3 ºC. ^{1}H-NMR (600 MHz, $CDCl_3$) δ(ppm) 8.122 (1H, d, *J* = 16.2 Hz, H-β), 8.079 (1H, d, *J* = 15.6 Hz, H-β'), 8.068 (1H, d, *J* = 6.6 Hz, H-3), 7.738 (1H, d, *J* = 7.2 Hz, H-6), 7.678 (1H, t, *J* = 15.0 Hz, H-4), 7.611 (1H, d, *J* = 6.0 Hz, H, H-6'), 7.555 (1H, t, *J* = 15.6 Hz, H-5), 7.390 (1H, t, *J* = 16.2 Hz, H-4'), 7.228 (1H, d, *J* = 16.2 Hz, H-α), 7.000 (1H, t, *J* = 10.8 Hz, H-5'), 6.961 (1H, d, *J* = 15.6 Hz, H-α'), 6.949 (1H, d, *J* = 8.4 Hz, H-3'), 3.933 (3H, s, 2'-OCH_3). ESI-MS m/z: 310.3 $(M+H)^+$.
