# Supplementary material for: Anti-inflammatory effects of novel curcumin analogs in experimental acute lung injury
Source: Respir Res. 2015 Mar 24;16(1):43. doi: 10.1186/s12931-015-0199-1 (PMC4391684; doi:10.1186/s12931-015-0199-1)
Supplement: Additional file 3: Figure S1. — The viability of mouse peritoneal macrophages was detected through an MTT assay after treatment active analogs for 24 h. Figure S2. The compounds stability was described by the curve which is consisted of absorbance at various optical density (250-600 nm) at the time 0, 0.5, 1, 2, 6, 12, and 24 h. [file 12931_2015_199_MOESM3_ESM.doc]

Additional File 3

**Anti-inflammatory effects of novel curcumin analogs in experimental acute lung injury**

Yali Zhang^1,2,#^, Dandan Liang^1,#^, Lili Dong^3^, Xiangting Ge^3^, Fengli Xu^3^, Yuanrong Dai^3^, Peng Zou^2^, Shulin Yang^2,*^, Guang Liang^1,*^

1. Chemical Biology Research Center at School of Pharmaceutical Sciences, Wenzhou Medical University, Wenzhou, Zhejiang 325035, China

2. School of Environmental and Biological Engineering, Nanjing University of Science and Technology, Nanjing, Jiangsu 210094, China

3. The 2^nd^ Affiliated Hospital, Wenzhou Medical University, Wenzhou, Zhejiang 325035, China

**Figure S1.** The viability of mouse peritoneal macrophages was detected through an MTT assay after treatment active analogs for 24 h.

**Figure S2.** The compounds stability was described by the curve which is consisted of absorbance at various optical density (250-600 nm) at the time 0, 0.5, 1, 2, 6, 12, and 24 h..
